# Data on association between QRS duration on prehospital ECG and mortality in patients with confirmed STEMI

**DOI:** 10.1016/j.dib.2017.08.051

**Published:** 2017-09-05

**Authors:** Rikke Hansen, Martin Frydland, Ole Kristian Møller-Helgestad, Matias Greve Lindholm, Lisette Okkels Jensen, Lene Holmvang, Hanne Berg Ravn, Jesper Kjærgaard, Christian Hassager, Jacob Eifer Møller

**Affiliations:** a Department of Cardiology, Odense University Hospital, Sdr Boulevard 29, DK 5000 Odense C, Denmark; b The Heart Center, Copenhagen University Hospital Rigshospitalet, Blegdamsvej, DK-2100 Copenhagen, Denmark

## Abstract

*Data presented in this article relates to the research article entitled “*Association between QRS duration on prehospital ECG and mortality in patients with suspected STEMI” (Hansen et al., in press) [1].

Data on the prognostic effect of automatically recoded QRS duration on prehospital ECG and presence of classic left and right bundle branch block in 1777 consecutive patients with confirmed ST segment elevation AMI is presented. Multivariable analysis, suggested that QRS duration >111 ms, left bundle branch block and right bundle branch block were independent predictors of 30 days all-cause mortality. For interpretation and discussion of these data, refer to the research article referenced above.

Specifications Table

**Table:** 

Subject area	*Health science*
More specific subject area	*Cardiology*
Type of data	*Table, figures*
How data was acquired	*Prospective observational*
Data format	*Analyzed*
Experimental factors	*Duration of QRS interval in ECG.*
Experimental features	*In consecutive patients with confirmed STEMI were prospectively included. QRS duration was registered from automated QRS measurement on the prehospital ECG that was send to the STEMI center where treatment was decided. Patients were divided according to quartiles of QRS duration. Primary endpoint was all-cause 30-day mortality. Predictors of all-cause mortality were assessed using Cox proportional hazards analysis.*
Data source location	*Copenhagen and Odense, Denmark*
Data accessibility	*The data are available with this article*

**Value of the data**

• *Data show that short term mortality is significantly higher in patients with suspected STEMI compared to those with confirmed STEMI*
• *QRS duration is an independent predictor of short term mortality in patients with confirmed STEM*

## 1 Data

QRS duration has previously shown association with mortality in patients with acute myocardial infarction treated with thrombolytics. Less is known of the prognostic value of QRS duration on prehospital ECG in patients with STEMI in an contemporary population. These data are based on prospective assessment of all patients with confirmed STEMI where an prehospital ECG was available that were referred to two high volume primary PCI centers in Denmark [1].

## 2 Experimental design, materials and methods

### 2.1 Study population

**Fig. 1:**
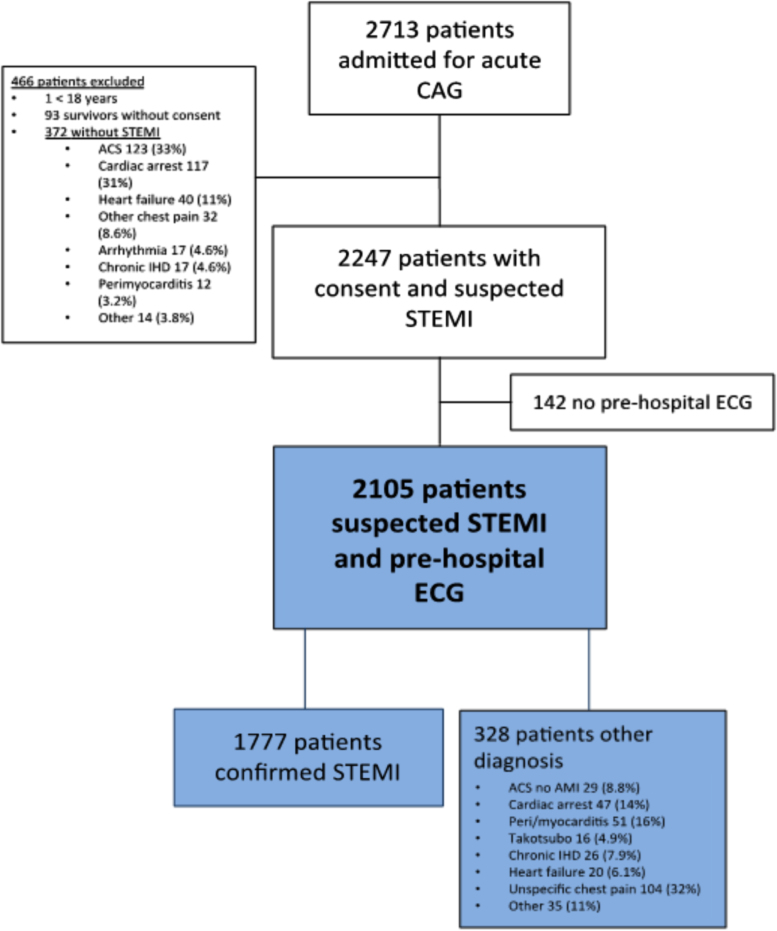
Consort diagram describing selection of patients. ACS denotes acute coronary syndrome; ECG denotes electrocardiogram; NSTEMI denotes non-ST segment elevation myocardial infarction; OHCA denotes out of hospital cardiac arrest; AMI denotes acute myocardial infarction; STEMI denotes ST segment elevation myocardial infarction.

Patients admitted with suspected STEMI were consecutively enrolled at the Heart Center, Rigshospitalet, Copenhagen from March 2015 to March 2016 and at the Department of Cardiology at Odense University Hospital Denmark from October 2015 to August 2016. Patients were excluded, if prehospital ECG was determined without significant ST segment elevation. Further, self-presenters and patients without prehospital ECG were excluded (Fig. 1).

### 2.2 Pre-hospital ECG

Standard 12 lead ECG was recorded by EMS using a LIFEPAK® 15 monitor/defibrillator (Physio-Control, Inc. Redmond, WA, USA) at a paper speed of 25 mm/s. All prehospital ECGs were later accessed by the medical record system and the automatically measured QRS duration was registered for this study together with presence of RBBB or LBBB. STEMI was identified on ECG as ST-segment elevation at the J-point ≥0.1 mV (mV) in two contiguous leads, other than in leads V2−V3 where following cut-points applied: ≥0.2 mV in men ≥40 years, ≥0.25 mV in men <40 years and ≥0.15 mV in women. 13 Final diagnosis was based on admission coronary angiography and evaluation of biomarkers of myocardial damage (high sensitive troponins) [2].

### 2.3 All-cause mortality

All-cause mortality was assessed using the Danish Civil Registration System, where all Danish citizens are recorded with a unique 10-digit personal number and where all deaths in Denmark are registered within 2 weeks. Initial follow up began on the date of admission. Follow-up of patients continued until date of death, or October 30th, 2016. Primary endpoint was all cause 30-day mortality.

### 2.4 Statistics

Data was analyzed using SPSS (IBM Statistics, Version 21.0). QRS duration was not Gaussian distributed thus the cohort was divided into quartiles of QRS duration (<89 ms, 89–98 ms, 99–111 ms and >111 ms).

**Fig. 2:**
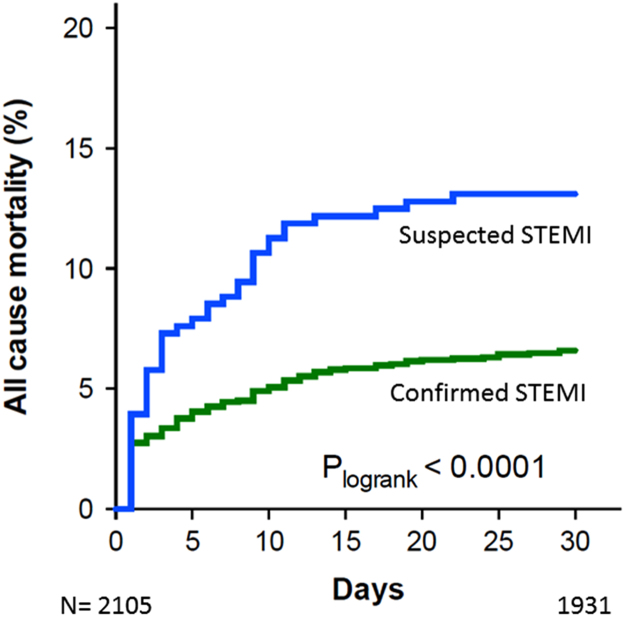
Survival in patients with suspected STEMI where the diagnosis was confirmed (blue) and where STEMI diagnosis was rejected.

**Fig. 3:**
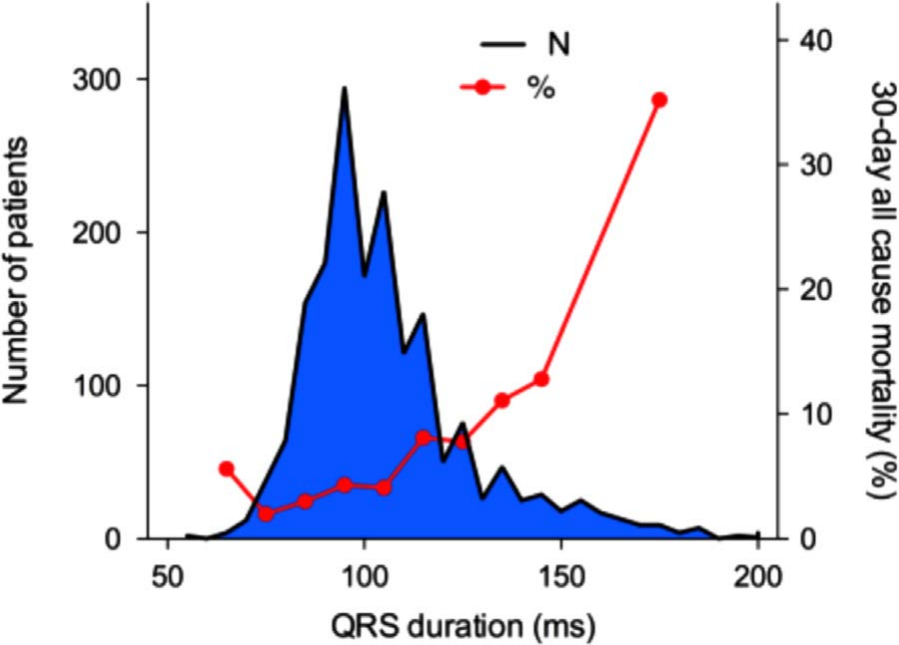
Histogram of number of patients, according to automatically measurement of QRS duration on prehospital ECG. Red line represents 30-day all-cause mortality according to QRS duration.

**Fig. 4:**
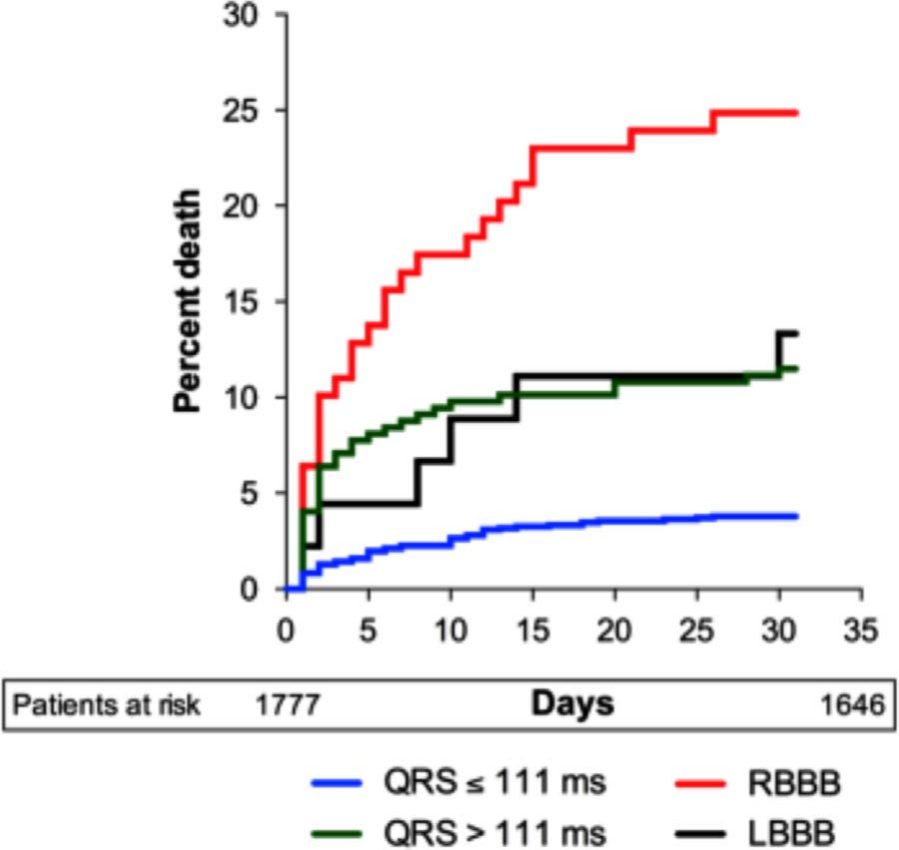
Kaplan Meier survival curve, in patients with suspected ST segment elevation myocardial infarction categorized according to QRS duration and bundle branch block on prehospital electrocardiogram.

All-cause mortality was assessed using Cox proportional hazard model to determine the association of QRS duration with mortality. The Kaplan Meier estimate of all-cause mortality was then calculated and plotted according to quartiles of QRS duration. For Cox proportional hazards analyses QRS duration was analysed as a categorical variable with the first quartile (<89 ms) serving as reference. In the Cox analysis patients with LBBB and RBBB were analysed separately, accordingly the group designated QRS duration >111 ms constituted patients with QRS duration 112–120 ms and those with unspecific conduction delay. In order to assess the value of QRS duration in prehospital triage, a Cox model based on prehospital variables (age, gender, systolic blood pressure, history of diabetes mellitus, previous myocardial infarction and whether the patient had suffered OHCA) was made. A two sided *p*-value <0.05 was considered statistically significant (Table 1 and Fig. 2, Fig. 3, Fig. 4)

**Table 1:** Cox regression model in patients with confirmed STEMI, based on QRS duration on prehospital ECG.

	Model 1			Model 2			Model 3		
	HR	95% CI	P	HR	95% CI	P	HR	95% CI	P
QRS									
<89	1.0	–	–	1.0	–	–	1.0	–	–
89–98	1.52	(0.75−3.05)	0.24	1.65	(0.82−3.32)	0.16	1.74	(0.82−3.70)	0.15
99–111	1.45	(0.71−2.99)	0.31	1.63	(0.78−3.38)	0.19	1.48	(0.66−3.31)	0.34
>111^a^	4.20	(2.22−7.97)	<0.0001	4.60	(2.39−8.86)	<0.0001	3.42	(1.70−6.87)	0.0006
LBBB	4.80	(1.82−12.63)	0.002	4.37	(1.65–11.56)	0.003	3.46	(1.27−9.41)	0.02
RBBB	9.62	(4.96−18.64)	<0.0001	8.37	(4.24−16.55)	<0.0001	3.84	(2.30−10.16)	<0.0001
Age				1.03	(1.02−1.05)	<0.0001	1.06	(1.04−1.07)	<0.0001
Gender (male)				0.58	(0.39−0.86)	0.007	0.65	(0.43−0.98)	0.04
History of DM							0.99	(0.59−1.66)	0.97
Prehospital cardiac arrest							3.75	(2.42−5.82)	<0.0001
Systolic blood pressure							0.98	(0.97−0.98)	<0.0001

DM, diabetes mellitus. LBBB, left bundle branch block. RBBB, right bundle branch block.

a QRS duration >111 ms without LBBB or RBBB configuration.
